# Engineering and Monitoring 3D Cell Constructs with Time-Evolving Viscoelasticity for the Study of Liver Fibrosis In Vitro

**DOI:** 10.3390/bioengineering8080106

**Published:** 2021-07-27

**Authors:** Ludovica Cacopardo, Arti Ahluwalia

**Affiliations:** 1 Research Center ‘E. Piaggio’, University of Pisa, 56122 Pisa, Italy; arti.ahluwalia@unipi.it; 2 Department of Information Engineering, University of Pisa, 56122 Pisa, Italy; 3 Interuniversity Center for the Promotion of the 3Rs Principles in Teaching and Research (Centro 3R), Italy

**Keywords:** 3D cell culture, engineered gels, transglutaminase, bioreactors, liver, fibrosis, mechanical monitoring, time-evolving viscoelasticity

## Abstract

Liver fibrosis is generally associated with an over-production and crosslinking of extracellular matrix proteins, causing a progressive increase in both the elastic and viscous properties of the hepatic tissue. We describe a strategy for mimicking and monitoring the mechano-dynamics of the 3D microenvironment associated with liver fibrosis. Cell-laden gelatin hydrogels were crosslinked with microbial transglutaminase using a purpose-designed cytocompatible two-step protocol, which allows for the exposure of cells to a mechanically changing environment during culturing. A bioreactor was re-engineered to monitor the mechanical properties of cell constructs over time. The results showed a shift towards a more elastic (i.e., solid-like) behaviour, which is likely related to an increase in cell stress. The method effectively mimics the time-evolving mechanical microenvironment associated with liver fibrosis and could provide novel insights into pathophysiological processes in which both elastic and viscous properties of tissues change over time.

## 1. Introduction

The liver is one of the largest and metabolically important organs of the human body. In normal liver, extracellular matrix (ECM) is restricted to portal tracts, sinusoid walls, and central veins, providing scaffolding for the cells and maintaining their differentiated phenotype and functions. Fibrillar collagen (types I, III, and V) is mainly found in the portal tract and central vein wall, while type IV collagen contributes to the formation of a low-density membrane-like material along the sinusoid wall. Other liver ECM components include glycoproteins, such as laminin and fibronectin, and proteoglycans such as chondroitin sulphate and hyaluronic acid [1,2,3]. Given the high hydration and low-density of these polymers, hepatic tissue manifests a strong viscoelastic behaviour [4], which has been characterised with a variety of mechanical testing methods, such as bulk compression and indentation tests (e.g., stress relation, creep and dynamic mechanical analysis), magnetic resonance elastography (MRE) and ultrasound techniques [5]. Fibrosis emerges as a consequence of chronic liver injury, independent of its aetiology, which may include viral infections (e.g., hepatitis B and C), drug toxicity, alcohol abuse or metabolic dysfunction, such as obesity and diabetes, which can lead to the non-alcoholic fatty liver disease (NAFLD) [1,2,6,7]. Fibrotic conditions are generally associated with an over-production of ECM proteins, due to fibroblast hyperproliferation and differentiation into collagen-secreting myofibroblasts, and with lysyl-oxidase (LOX) upregulation, which promotes the formation of stable inter-molecular collagen crosslinks [3,8,9]. Healthy liver has been reported to have an elastic modulus (*E)* of around a few kPa, which increases up to 20 kPa in the last stage of fibrosis [7,10,11,12]. The increase in collagen also affects the viscosity of hepatic tissue, which in several studies was found to increase twofold [13,14]. These alterations provoke organ dysfunction, leading to diseases, such as portal hypertension and liver failure [9,15].

Advanced in vitro models (IVMs) able to recapitulate the liver microenvironment have been developed using different types of hydrogels with elastic moduli ranging from 0.4 to 22 kPa (e.g., Matrigel, polyacrylamide, collagen, alginate and agarose) [2,16,17,18,19]. However, despite the efforts towards the generation of mechano-mimetic liver IVMs, only a few examples of viscoelastic substrates capable of reproducing the ‘healthy-to-fibrotic’ transition can be found in the literature. For example, Guvendiren and colleagues, demonstrated that the elastic modulus of methacrylated hyaluronic acid (MeHA) gels can be varied from 2 kPa (‘healthy stiffness’) to 24 kPa (‘fibrotic stiffness’) after ultra-violet (UV) light exposure [20]. Similarly, Hui et al. were able to increase both the *G*′ and *G*″ of Norbornene-HA gels using UV light, passing from a soft gel with a *G*′ around 0.5 kPa to a stiff one with a *G*′ around 5 kPa [21]. The relaxation behaviour of the norbornene-polyethylene glycol (PEG) thiol gels was also modulated thanks to UV exposure [22]. To avoid UV cytotoxic effects, Caliari and co-workers fabricated soft MeHA gels (*E* = 1.75 kPa) which can be crosslinked in situ with visible blue light to obtained stiffer gels (*E* = 33 kPa) with a concomitant increase in both storage (*G*′) and loss (*G*″) shear moduli [23]. In addition, enzymatic crosslinking with transglutaminase, an enzyme found in several microorganisms, vegetal and animal organisms (including humans), was used to modulate ‘on-demand’ the viscoelastic properties of glutaraldehyde crosslinked gelatin gels [24]. All these studies are limited to 2D cell culture, although the relevance of 3D culture condition has been proven in many applications [25,26].

Based on these considerations, we developed a strategy to encapsulate hepatocytes in gelatin-based hydrogels with time-evolving viscoelastic properties [24]. The strategy is based on a two-step crosslinking process using microbial transglutaminase (mTG), which catalyses the formation of covalent bonds between glutamine and lysine amides. In the first step, mTG was used to stabilise the gels and then applied exogenously to modulate their viscoelastic behaviour [27,28]. Gelatin, a highly hydrophilic polymer derived from collagen, was chosen for its biocompatibility and viscoelastic nature which matches that of the ECM. This biopolymer is able to form physically (reversible) or more stable chemically (permanent) crosslinked hydrogels thanks to temperature variations or the formation of covalent bonds respectively [29,30]. In the case of enzyme-mediated covalent crosslinks, the reaction kinetics depend on the polymer structure and composition, the ratio between reactant and enzyme concentration and environmental conditions, such as temperature and pH [27,31]. Consequently, the evolution of the gels’ mechanical properties can be modulated by controlling any of these reaction parameters.

The in situ monitoring of the mechanical properties during cell culture is fundamental for understanding how cells and the materials they are seeded on change their mechanical behaviour over time. In this direction, different bioreactors with integrated mechanical actuation and sensing have been developed [32,33,34,35,36]. The systems able to perform unconfined bulk compression (i.e., the most suitable testing strategy for characterising hepatic cell constructs) are mainly based on electromagnetic actuators [37,38,39,40,41,42], hydraulic and vacuum pumps [43,44,45] and on air pressure regulators [46]. Sensing can be performed thanks to load cells or thin film force sensor [37,39,40,41,42], optical encoders [38], including laser sensors [43], ultrasound imaging [44], linear variable displacement transducers (LVDTs) [45] or Hall effect sensors [46]. These systems generally allow for testing multiple samples; however, many of them are unsuitable for the real-time testing of soft tissue constructs, as required for liver IVMs. In order to monitor the viscoelastic behaviour of these samples over time, without damaging cells or irreversibly deforming the scaffolds, mechanical tests should be performed within their linear viscoelastic region (LVR). Therefore, a low force version of the MechanoCultureTR (MCTR) bioreactor (CellScale, Waterloo, ON, Canada) was developed in collaboration with the company to match these requirements and allow for the monitoring of the cell-laden mTG-gelatin hydrogels.

## 2. Materials and Methods

### 2.1. Re-Engineering the MCTR Bioreactor

**Figure 1:**
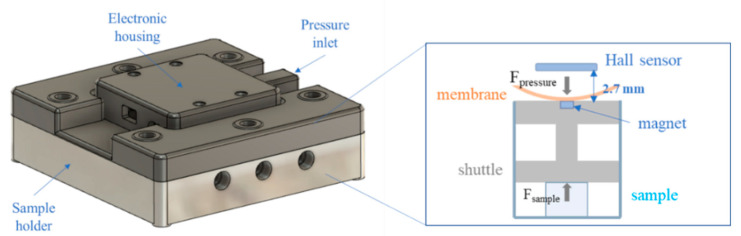
Technical drawing of the low-force MCTR bioreactor (**right**) and representation of one well (**left**).

The MCTR bioreactor (CellScale, Waterloo, ON, Canada) allows for the culturing of cell constructs in a mechanically active environment. It is composed of 9 wells, each one containing a scaffold or cell-laden construct and a shuttle with a cylindrical magnet (diameter = 10 mm, height = 2.5 mm) on top (Figure 1). Pressure applied to the chamber above the wells deforms the membrane that separates the two compartments. A pressure regulator is used to compress the samples with a known force or force rate, while the displacement is monitored using Hall effect sensors [46].

In its commercial version, the bioreactor is equipped with a 5–500 kPa pressure regulator with a resolution of 0.1 kPa that allows a force operation range from about 2 to 100 N. To enable low-force measurements (around few N), a 0–7 kPa pressure regulator (QPV1, Equilibar, Fletcher, NC, USA) with a resolution of 0.35 mPa was selected. In order to minimize the undesired pre-compression of the sample, a polylactic acid (PLA) shuttle and smaller magnets (2.5 × 1 mm) were used, resulting in a maximum weight of 15 mN. Owing to the weaker magnetic field, the MCTR top was modified to reduce the working distance (from 13 mm to 2.7 mm) between magnets and sensors, which were re-calibrated accordingly (Figure 1). Finally, the 60A food-grade silicone membrane (thickness = 0.8 mm) was replaced with a 10A membrane with the same characteristics (McMaster-Carr, Elmhurst, IL, USA). Table 1 summarises the technical modifications made to the bioreactor.

### 2.2. Gel Fabrication and Cell Encapsulation

**Figure 2:**
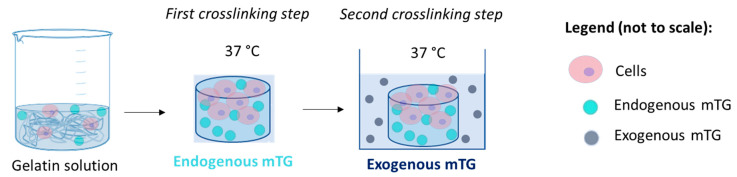
Preparation of the cell-laden gel, outlining the two crosslinking steps based on endogenous and exogenous mTG.

Modified Eagle Medium (MEM) with 10% FBS, 1% L-Glutamine and 1% Penicillin/Streptomycin was used for the preparation of the gels and for cell culture. All reagents were purchased from Sigma-Aldrich (Milan, Italy). HepG2 cells (3 × 10^5^ cells/mL) were encapsulated in 5% *w/v* gelatin (Type A, G2500, Sigma-Aldrich) pre-crosslinked with 5 Units/gram (U/g) mTG. This line derived from human carcinoma is one of the most commonly used hepatic cell lines and it is known to expresses several liver-specific genes in culture [16]. Gelatin powder was sterilised by UV exposure (around 30 min) and dissolved in warm culture medium (around 50 °C) to obtain a 10% gelatin solution, then kept at 37 °C, while the enzyme was diluted in the cell culture medium at a concentration of 10 U/g at 37 °C and then sterilized by filtration. For the controls, the two solutions were mixed together, obtaining the final mTG-gelatin solution. To obtain the cell-laden gels, the cells were suspended in the sterile mTG solution and mixed with the gelatine solution. Finally, the solutions were cast into custom polydimethylsiloxane (PDMS) moulds (8 mm high, 13 mm diameter) and incubated for 8 h at 37 °C. As shown in Figure 2, this first crosslinking step thus contains endogenous mTG, which is necessary for the stability of gelatin-based gels at 37 °C. In the second crosslinking step, which allows for the modulation of gel viscoelasticity over time, the gels were submerged in medium containing 100 U/g of exogenous mTG and left in culture until day 11. The mTG-containing medium was replaced every 3 days.

### 2.3. Gel Water Content and Degradation

The bicinchoninic acid (BCA) assay (71285 Millipore, Burlington, MA, USA) was used to quantify the relative protein content released from the G and G-ex gels. Samples were collected from the supernatant at day 1, 7 and 11 and absorbance was read at a wavelength of 565 nm with a spectrophotometer (VICTOR, PerkinElmer, Waltham, MA, USA). Gel degradation was calculated as the percentage of protein mass released with respect to the initial mass of gelatin and mTG in the samples.

G and G-ex gels were weighed at day 1, 7 and 11 (Radwag AS 220/C/2—Radom, Poland) to evaluate hydrogel water content, which was calculated as the difference between the mass of the hydrated gel and of the dry protein mass, normalized with respect to the initial mass of the gel. For each sample, the effective dry protein mass was calculated, considering the protein loss.

### 2.4. Mechanical Testing in the Low-Force MCTR Bioreactor

**Figure 3:**
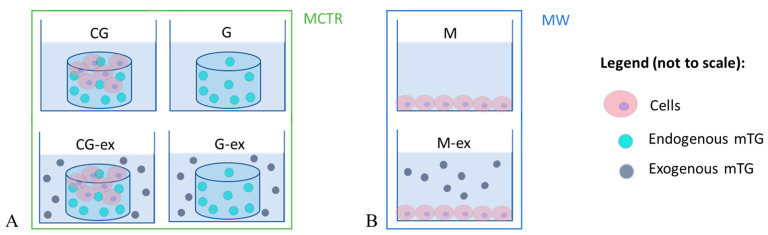
Summary of the experimental setup and acronyms: (**A**) samples tested in the bioreactor, i.e., cell-laden gels (CG), cell-laden gels with exogenous mTG (CG-ex), gels (G) and gels with exogenous mTG (G-ex); (**B**) monolayers in multiwell plates cultured in the absence (M) and in the presence of exogenous mTG (M-ex).

The mechanical properties of cell-laden gels cultured in the presence (CG-ex) or in the absence (CG) of exogenous mTG were monitored in the bioreactor. Moreover, gels without cells were prepared following the same crosslinking steps, obtaining gels with (G-ex) and without exogenous mTG (G). Figure 3 summarises the experimental setup in the bioreactor along with the symbols used for each type of sample. Creep tests were performed under unconfined compression with a 0.04 N/s ramping phase, applying a 20 mN force step for 15 min. For all samples, the strain during the creep test was maintained below 10%, i.e., within the linear viscoelastic region for gelatin-based hydrogels [24,46]. The tests were repeated on days 1, 7 and 11 of culture.

**Figure 4:**
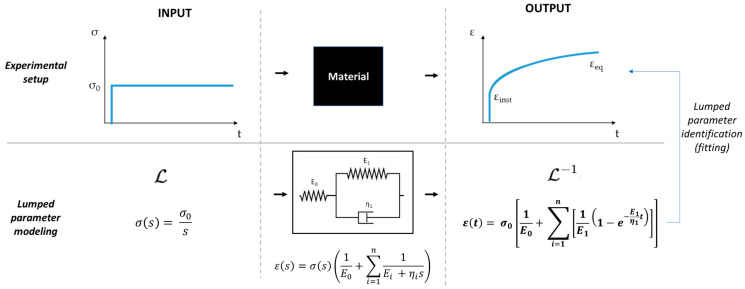
Experimental and data analysis workflow: the material under investigation interrogated with a step stress input produces a strain–time curve as output (creep). The strain–time equation obtained modelling the material with a one-element GV model is then fitted to the experimental curve to identify the lumped parameters, which characterise the material viscoelastic descriptors.

Creep data were converted into engineering strain–time (ε-t) curves (ε = ∆h/h_0,_ where ∆h is the gel deformation and h_0_ represents its initial height). Following the workflow reported in [46], the step response of a Generalized Voigt (GV) model (Figure 4), composed of a pure spring (E_0_) in series with one Voigt element (i.e., a parallel between a spring and a dashpot, $E_{1}$ and $\eta_{1}$), was fitted to the experimentally averaged ε–t curves obtained from 3 independent gels. Finally, the three descriptors—(i) the characteristic retardation time $\tau =\eta_{1}/E_{1}$, (ii) the instantaneous elastic modulus $E_{\mathrm{inst}}=E_{0}$ and (iii) the equilibrium elastic modulus as $E_{\mathrm{eq}}=\frac{E_{0}E_{1}}{E_{0}+E_{1}}$—were calculated. The viscoelastic descriptors are reported as mean and standard error of the mean (SEM).

### 2.5. Cell Viability and Staining

The gels were observed under a microscope (Olympus—Tokyo, Japan). Cell viability was assessed with Alamar Blue (Sigma-Aldrich—Milan, Italy) on days 0, 4, 7 and 11. A 10% resazurin solution was prepared in complete culture media and incubated with the hydrogels for 6 h at 37 °C. Then, three media samples of 100 µL were collected for each gel and analysed in a fluorescence spectrophotometer (VICTOR, PerkinElmer, Waltham, MA, USA) using an excitation wavelength of 490 nm and an emission wavelength of 610 nm. To test for mTG cytotoxicity, cell monolayers were cultured in the presence (M-ex) or in the absence (M) of 100 U/g mTG in the media (Figure 2B), and viability was measured at day 0 and 11.

At the end of culture, hydrogels were cut into smaller slices (around 1 mm thick), fixed with 4% paraformaldehyde (PFA) and permeabilized with 0.1% Triton. Cell nuclei were stained with DAPI and actin with green Alexa fluor 488-conjugated phalloidin (ThermoFisher, Waltham, MA, USA). Images were acquired with a confocal microscope (Nikon A1, Tokio, Japan).

### 2.6. Statistical Analysis

Statistical differences were tested using (i) two-way Analysis of Variance (ANOVA) followed by Tukey’s Multiple Comparison Test for gel degradation and water content analysis of the G and G-ex and for viability of the CG and CG-ex; (ii) one-way ANOVA followed by Sidak’s Multiple Comparison Test for the viscoelastic descriptors of the G, CG, G-ex and CG-ex; (iii) the Studentʹs *t*-test for the M and M-ex viability. All experiments were performed in triplicate (number of independent experiments, n = 3) and all statistical analyses were performed using GraphPad Prism (GraphPad Software, San Diego, CA, USA), setting significance at *p* < 0.05.

## 3. Results

As shown in Table 2, after re-engineering, the requirements for low-force viscoelastic monitoring of cell constructs were achieved.

**Figure 5:**
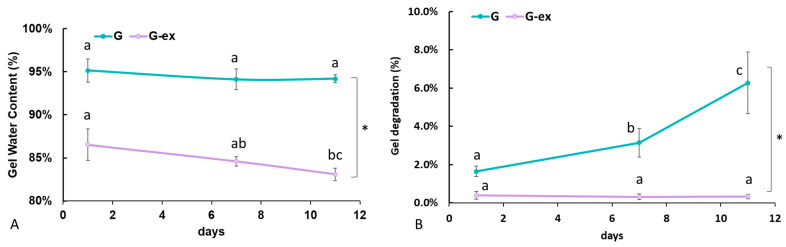
(**A**) Water content, calculated as the percentage of the total initial mass of the gel; (**B**) Gel degradation, expressed as the percentage of protein content in the supernatant with respect to the initial protein content (* = *p* < 0.5). For each gel type, different letters indicate significant differences over time (*p* < 0.5).

Figure 5 reports the variations in gel water content and protein degradation over time. The initial gel water content was significantly higher for the G with respect to the G-ex (*p* < 0.0001, Figure 5A). In the G, water content did not vary appreciably over time but, in the G-ex samples, a significant (*p* = 0.0069) decrease in water content is observed as a function of time, reflecting gel shrinking, which related to exogenous mTG crosslinking. However, G gel degradation was significant over time (*p* < 0.0001, Figure 5B). On the contrary, G-ex degradation did not vary over time and was significantly lower with respect to G (*p* < 0.0001), suggesting that exogenous mTG effectively improves the gel’s resistance to hydrolytic attack.

**Figure 6:**
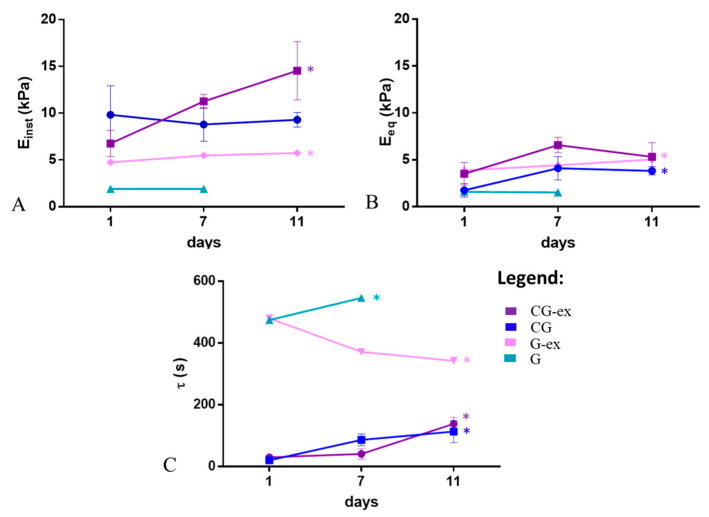
Viscoelastic descriptors over time measured in the bioreactor: (**A**) instantaneous, $E_{\mathrm{inst}}=E_{0}$ and (**B**) equilibrium modulus, $\left(E_{\mathrm{eq}}=\frac{E_{0}E_{1}}{E_{0}+E_{1}}\right)$, and (**C**) retardation time, $\tau =\eta_{1}/E_{1}$ (* = *p* < 0.05).

Figure 6 shows the viscoelastic characterisation. In the G-ex samples, with exogenous mTG, a significant increase in both E_inst_ and E_eq_ (*p* < 0.0001) and a decrease in τ was observed (*p* = 0.0003), clearly due to the activation of mTG. On the contrary, for the G samples without exogenous mTG, the moduli remained almost constant (E_inst_ ~1.9 kPa and E_eq_ ~1.5 kPa) and τ increased over the first 7 days, reflecting an increase in their liquid-like behaviour. In fact, we were unable to conduct further mechanical tests on the 11th day of culture as the gels were unable to retain their shape. The BCA data (Figure 5B) confirm that these gels are prone to degradation even though they undergo crosslinking. In the presence of cells, CG-ex and CG samples generally present higher moduli and lower retardation times with respect to G-ex and G samples. Furthermore, CG-ex gels have a higher modulus with respect to CG, clearly due to the exogenous mTG crosslinking. In particular, for the CG-ex samples, we observed a significant increase in E_inst_ (*p* = 0.0094) and τ (*p* = 0.0080). Finally, in the CG gels, E_inst_ does not vary over time, while E_eq_ presents an increasing trend from day 1 to day 7 (*p* = 0.0311) and τ significantly increases (*p* = 0.0003).

**Figure 7:**
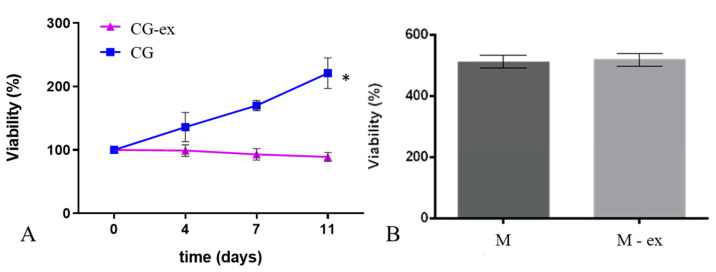
Cell viability in the CG and CG-ex gels over time (**A**) and in monolayer controls in the absence (M) and in the presence of exogenous mTG (M-ex) at the 11th day of culture (**B**). Data are normalised with respect to day 0 (* = *p* < 0.05).

As shown in Figure 7A, while the cell viability in the CG gels increases with time (*p* = 0.0054), we did not observe significant changes in viability over the 11 days of culture in the CG-ex samples (i.e., in the presence of exogenous mTG). To assess the cytotoxicity of exogenous mTG, monolayers of HepG2 cells were exposed to media containing the same concentration of enzyme as the G-ex and CG-ex gels. We did not observe significant differences in viability between cells cultured in the presence (M-ex) or absence (M) of exogenous mTG (Figure 7B).

**Figure 8:**
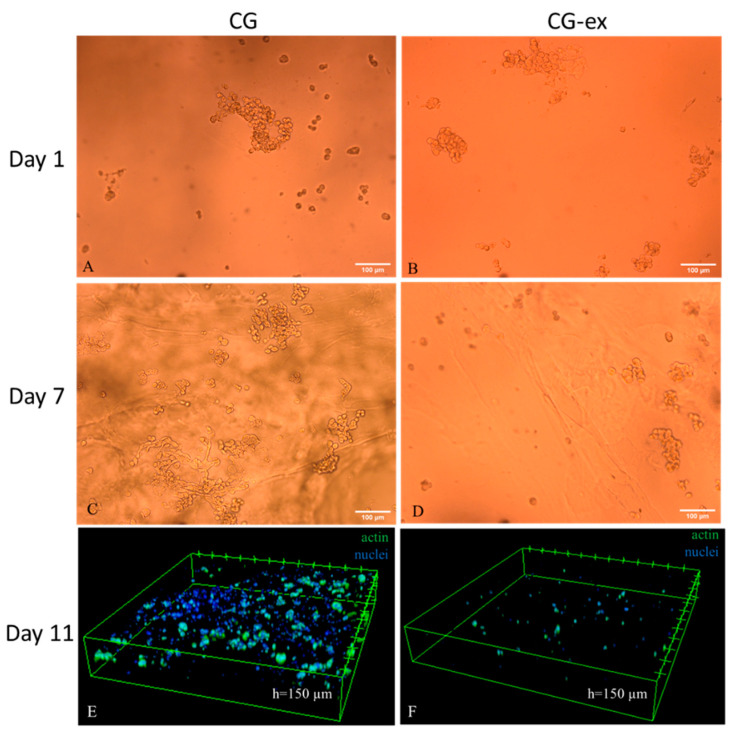
Cell distribution in the cell-laden gels: 10X bright field images of the CG and CG-ex samples on days 1 ((**A**) and (**B)** respectively) and 7 ((**C**) and (**D**) respectively); 10X confocal acquisition on day 11 of the CG (**E**) and CG-ex (**F**). In panels E and F, cells are stained with DAPI (nuclei) and Alexa fluor 488-conjugated phalloidin (actin).

Finally, in Figure 8, bright field and confocal images indicate that, in the CG gels, there are a higher number of cells forming clusters, while very few cells are present in the CG-ex gels.

## 4. Discussions

The work is focused on the development of a strategy to engineer and monitor the mechanical properties of gels able to mimic the progression of fibrosis over time. First, the MCTR bioreactor was re-engineered to monitor the mechanical behaviour of cell constructs during culture. In particular, the stimulation range within 0.2–1.4 N and a resolution of 0.7 mN allowed for the mechanical testing of the same samples over time without damaging the cells or the material. We then describe a fully biocompatible two-step crosslinking method for the generation of cell-laden hydrogels with time-evolving viscoelasticity [24]. The hydrogels were composed of gelatin crosslinked with endogenous mTG (G), which could be subsequently stiffened by adding a solution of exogenous mTG (G-ex). Preliminary cell tests were performed to assess the cytocompatibility of the method.

The increase in E_inst_ and E_eq_ and corresponding decrease in the retardation time in the G-ex gels suggest that the exogenous mTG diffuses inside the gels and reacts with gelatin, inducing gel crosslinking, which results in stiffer and more elastic, or solid-like, gels over time. This observation is further confirmed by the gel shrinking and its resistance to degradation. Thus, the second crosslinking step is able to mimic the concomitant increase in liver stiffness and viscosity related to fibrotic processes associated with an increase in both ECM amount and crosslinking [3,8,9].

On the contrary, the gels without exogenous mTG (G) became more viscous or liquid-like over time, as observed from the significant decrease in τ and from the high degradation rate. Indeed, it was not possible to test the gels at day 11 as they were unable to retain their shape under compression. This suggests that the crosslinking with endogenous mTG is not sufficient to overcome competing phenomena, such as hydrolysis [24].

Comparing the cell-laden gels (CG and CG-ex) to the gels without cells (G and G-ex), we note that the cells significantly affect the viscoelastic properties of the materials. In particular, the lower retardation time of both CG and CG-ex indicates that the presence of cells results in a shift towards a more solid-like behavior. Indeed, cells are likely to produce their own matrix, which counteracts gel hydrolysis [47]. However, the fact that the retardation times gradually increase in both cell-laden gels could be related to cell proteolytic activity, which overrides endogenous mTG crosslinking. Nevertheless, the effects of exogenous mTG activity are still evident, since E_inst_ and E_eq_ significantly increase in CG-ex with respect to the CG samples.

In the absence of exogenous mTG, cell viability steadily increased over time, more than doubling after 11 days in the CG. On the contrary, cell viability did not increase in the presence of the exogenous enzyme (CG-ex). Confocal and brightfield images suggest that this is likely related to a higher cell proliferation in the CG gels with respect to the CG-samples. Since cell viability in the monolayers cultured with and without exogenous mTG (M-ex and M) was identical, we can assume that exogenous mTG has no cytotoxic effects at the concentrations used (100 U/g). Thus, the lower cell viability and proliferation in the CG-ex samples could be related to the mechanical alteration of the cellular environment. However, it should be underlined that crosslinking not only affects the mechanical properties, but also gel permeability, likely limiting oxygen and nutrient diffusion [48]. In the case of a 3D culture system, as well as in vivo, these effects are always related, and their decoupling is still a challenge.

In the future, the use of ‘more mechanosensitive’ cells, such as primary hepatic cells, and more specific cell analysis (e.g., albumin and urea secretion) will be important to refine the model [1,10,49].

## 5. Conclusions

In conclusion, using a two-step gelatin crosslinking strategy based on mTG, we developed a novel strategy for reproducing the mechanical alterations of the liver microenvironment, related to fibrosis development and monitoring them in vitro. Unlike other methods, which include crosslinking steps based on cytotoxic chemical crosslinkers [24], the biocompatibility of the enzyme allows for cell encapsulation and culture in 3D conditions. Moreover, the MCTR bioreactor allowed us to assess the time-evolving viscoelastic properties of the cell constructs.

This study represents a first step towards the development of physiologically relevant models, which are useful for understanding the mechanobiology of fibrosis. Further developments and applications are in the study of antifibrotic drugs that intervene in the fibrotic process a priori instead of acting a posteriori on fibrotic tissues or on inhibiting post fibrotic symptoms.

Enzymatic crosslinking can be implemented with any material possessing free amine groups (e.g., collagen or ECM derived materials). In addition, as it recapitulates pathophysiological processes, in which both elastic and viscous properties increase over time, this approach can be implemented to model other phenomena, such as embryogenesis and growth, in which the complex orchestration of cell division, differentiation and ECM synthesis results in an increase in tissue stiffness, density and crosslinking along with a decrease in fluidity and time dependency [50,51].

## Figures and Tables

**Table 1:** Differences between the low-force and commercial MCTR bioreactor.

	Low-Force Version	Commercial Version
**Pressure regulator**	Range: 0–7 kPa Resolution: 0.35 mPa	Range: 5–500 kPa Resolution: 0.1 kPa
**Membrane Hardness**	10A	60A
**Magnet dimensions** **(diameter × height)**	2.5 × 1 mm	10 × 2.5 mm
**Shuttle material**	PLA	Stainless steel

**Table 2:** Comparison between the low-force and commercial MCTR bioreactor performance.

	Low-Force Version	Commercial Version
**Force range**	0.02–1.4 N	2–100 N
**Force resolution**	0.7 mN	0.02 N
**Shuttle weight**	15 mN	133 mN
**Viscoelastic testing**	yes	yes [46]

## Data Availability

Data will be available under reasonable request.
